# Tailored Anticoagulation for Thromboembolic Risk Reduction in Paroxysmal Atrial Fibrillation

**DOI:** 10.19102/icrm.2018.090404

**Published:** 2018-04-15

**Authors:** Jason D. Matos, Jonathan W. Waks, Peter J. Zimetbaum

**Affiliations:** ^1^Harvard-Thorndike Electrophysiology Institute, Beth Israel Deaconess Medical Center, Harvard Medical School, Boston, MA, USA

**Keywords:** Anticoagulation, atrial fibrillation, stroke, thromboembolism

## Abstract

Atrial fibrillation (AF) is the most common sustained cardiac arrhythmia, affecting up to six million people in the United States and more than 35 million individuals worldwide. Thromboembolism, including stroke, represents the most common AF-related morbidity and mortality and data indicate that anticoagulation can mitigate this risk by 65%. Our understanding of thromboembolism in AF, however, remains incomplete, and the mechanisms by which AF increases thromboembolic risk are areas of ongoing investigation and debate. Current guidelines do not differentiate between the frequency and duration of AF episodes (AF burden) when selecting which patients with AF should be treated with anticoagulation for thromboembolic risk reduction. Recent data, primarily using cardiac implantable electronic devices (CIEDs) such as pacemakers, implantable cardioverter-defibrillators, and implantable loop recorders, however, have challenged this longstanding notion that AF burden does not influence thromboembolic risk. Continuous and automated cardiac rhythm monitoring via CIEDs with accurate and rapid acquisition and transmission of rhythm data also affords the opportunity to study the relationship between AF burden and thromboembolism and novel ways to reduce thromboembolic risk while minimizing the risk associated with chronic anticoagulation use. This manuscript will review the associations between subclinical, CIED-detected atrial arrhythmias and thromboembolic events. It will also discuss the emergence of “tailored anticoagulation,” an anticoagulation strategy wherein CIEDs and remote AF monitoring are employed to allow dynamic administration of oral anticoagulation only around episodes of AF, and the holding of anticoagulation during prolonged periods of sinus rhythm when the thromboembolic risk associated with AF is presumably very low.

## Introduction

Atrial fibrillation (AF) is the most common sustained cardiac arrhythmia, affecting up to six million people in the United States^1^ and more than 35 million individuals worldwide.^2^ The incidence and prevalence of AF are increasing rapidly and, by 2030, it is expected that up to 12 million people in the US will have AF.^3^ Thromboembolism, including stroke, represents the most common AF-related morbidity and mortality,^4^ and data indicate that anticoagulation can mitigate the risk of stroke by 65%.^5^ Our understanding of thromboembolism in AF, however, remains incomplete, and the mechanisms by which AF increases thromboembolic risk are areas of ongoing investigation and debate.^6^ Current guidelines also do not consider the frequency and duration of AF episodes (AF burden) when deciding which patients with AF should be treated with anticoagulation for thromboembolic risk reduction.^7^ Recent data primarily using cardiac implantable electronic devices (CIEDs) such as pacemakers, implantable cardioverter-defibrillators (ICDs), and implantable loop recorders (ILRs) have challenged the longstanding idea that AF burden does not influence thromboembolic risk. Continuous and automated cardiac rhythm monitoring with accurate and rapid acquisition and transmission of rhythm data via CIEDs also affords the opportunity to study the relationship between AF burden and thromboembolism and novel ways to reduce thromboembolic risk while minimizing the risk associated with chronic anticoagulation use.

## Origins of the associations between atrial fibrillation and thromboembolism

William Wood first described a left atrial thrombus in a patient with mitral stenosis more than 200 years ago.^8^ A clearer association between what was at the time termed “auricular fibrillation” and atrial thrombosis was not appreciated, however, until the 1930s.^9^ Although a clear association between AF and thromboembolism was observed in patients with rheumatic heart disease and mitral stenosis,^10^ the link between AF and thromboembolism in patients without rheumatic heart disease was not well-recognized until a seminal analysis of the Framingham Heart Study was conducted in the 1970s, which demonstrated that chronic AF without rheumatic heart disease was associated with a more than fivefold increase in the risk of stroke.^11^ In the 1980s, a link between paroxysmal AF and increased rates of thromboembolic complications in chronic versus paroxysmal AF was observed.^12^ Multiple large clinical trials have since confirmed a clear association between AF and thromboembolism in patients with paroxysmal, persistent, or chronic AF and a reduction in thromboembolic complications with the use of anticoagulation.^5,13–17^ Yet, despite the wealth of evidence supporting an association between AF and thromboembolism, the actual mechanism(s) of stroke and systemic embolism in patients with AF remain unclear and under investigation.^6^

## Current decision support for which patients should receive anticoagulation

The CHADS_2_ score, one of the most ubiquitous scoring systems in all of medicine, has long been the gold standard for assessing thromboembolic risk associated with AF and the need for anticoagulation.^18^ More recently, the updated CHA_2_DS_2_-VASc score has been validated and incorporated into the most recent AF guidelines of all major cardiovascular societies.^7,19^ Both scoring systems include the risk factors of congestive heart failure, hypertension, diabetes mellitus, and prior stroke or transient ischemic attack. However, the CHA_2_DS_2_-VASc score emphasizes the important relationship between age and thromboembolism, assigning two risk points for age ≥ 75 years and one risk point for age ≥ 65 years (versus one risk point for age ≥ 75 years in the original CHADS_2_ score). The CHA_2_DS_2_-VASc score also adds female gender and vascular disease as thromboembolic risk factors, although controversy exists about the relative risk associated with these risk factors in comparison with others (which are all assigned equal risk in the score). The main strength of the newer CHA_2_DS_2_-VASc score is its ability to identify a specific low-risk population (score: 0 points) who have a very low thromboembolic risk and who can reasonably be treated without systemic anticoagulation. Unfortunately, only 5% to 10% of the AF population (and no women with AF) meet this criteria.^20^

There is overall consensus that, in patients with a CHA_2_DS_2_-VASc score ≥ 2, systemic anticoagulation with an oral anticoagulant with either adjusted-dose warfarin or a new direct oral anticoagulant (DOAC) is indicated. For patients with a CHA_2_DS_2_-VASc score of 1, European guidelines recommend anticoagulation, whereas US guidelines suggest that either anticoagulation, aspirin use, or no anticoagulation are equally appropriate options. Controversy also exists regarding whether those with a CHA_2_DS_2_-VASc score of 2 due to certain risk factors require anticoagulation, because, although all risk factors in the score are given relatively equal weight (with the exception of age > 75 years and a history of stroke or transient ischemic attack), some studies have suggested that all risk factors may not be equal in terms of their influence on thromboembolic risk.^21,22^

## Atrial fibrillation burden and thromboembolic risk

Notably absent from the CHADS_2_/CHA_2_DS_2_-VASc scoring systems is the frequency and/or duration of AF episodes (AF burden). Furthermore, current guidelines do not consider AF burden when evaluating a patient’s risk of thromboembolism and their need for systemic anticoagulation. There is therefore inconsistency in the current AF guidelines, where the decisions to initiate and continue anticoagulation are independent of AF burden, but thromboembolic risk in the pericardioversion period is thought to remain relatively low for the first 48 hours.^7^ This “48-hour rule,” where it is acceptable to perform a cardioversion without first excluding a left atrial thrombus via transesophageal echocardiogram in the first 48 hours, however, is based on a relatively small, nonrandomized study,^23^ and no randomized controlled trial data exist to definitively support 48 hours as being a “safe” cutoff time period for thrombus formation. In fact, more recent data obtained from patients with pacemakers and ICDs that have the ability to precisely detect short, subclinical, and asymptomatic AF episodes have demonstrated that the risk of thromboembolism in AF begins to increase much earlier than at 48 hours after AF onset (see below).

It would seem logical that AF-associated intracardiac thrombus formation should be a time-dependent phenomenon. However, this has been difficult to prove due to the fact that episodes of AF can be asymptomatic and it can therefore be difficult to determine the exact onset of an AF episode by patient report. In fact, multiple studies have demonstrated that patient-reported symptomatic AF episodes are poorly correlated with device-detected AF episodes. In the Suppression of Paroxysmal Atrial Tachyarrhythmias (SOPAT) trial, only 46% of AF episodes detected on event monitors were symptomatic.^24^ Asymptomatic AF recurrences are even more common following catheter ablation for AF.^25,26^

Additionally, until recently, there were data showing that AF burden did not influence the risk of thromboembolism associated with AF. Older studies such as the AF Clopidogrel Trial with Irbesartan for Prevention of Vascular Events (ACTIVE W)^27^ and the Stroke Prevention in AF (SPAF) study^28^ found no significant difference in thromboembolic risk in patients with paroxysmal or chronic AF. Studies that have investigated rhythm control of AF, such as the AF Follow-up Investigation of Rhythm Management (AFFIRM) study, also demonstrated a significantly increased risk of stroke in patients who stopped anticoagulation despite being in sinus rhythm, although, in retrospect, this is likely secondary to the fact that antiarrhythmic therapy for AF is not 100% effective and many of these patients may have had asymptomatic AF that was not clinically detected.^29^ Multiple other observational studies have also found no significant differences in stroke rates between those with paroxysmal and persistent AF.^5,30^

However, all of these studies defined paroxysmal AF with surface electrocardiograms (ECGs) performed during physician visits that occurred months apart, and, as such, these studies therefore incompletely assessed AF burden and likely underestimated the true frequency and duration of AF episodes. These studies also suffer from limitations in statistical power; a large number of patients and events are needed to find a difference in thromboembolic events among those with paroxysmal and persistent/chronic AF. In fact, a more recent secondary analysis of the very large Apixaban for Reduction in Stroke and Other Thromboembolic Events in AF (ARISTOTLE) trial with more than 18,000 patients revealed a lower risk of thromboembolic events in patients with paroxysmal AF versus in those with persistent/permanent AF.^31^

## Remote monitoring to determine atrial fibrillation burden and stroke risk

**Figure 1:**
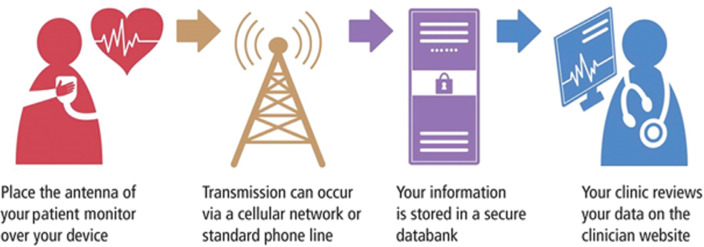
Remote transmission of arrhythmia data. Reproduced with permission from Zimetbaum et al.^52^

CIEDs, including pacemakers, ICDs, and ILRs, offer a precise method of defining AF burden that does not rely on patient-reported symptoms or frequency of surface ECGs. Although algorithms for AF detection are not perfect (they can result in both false-positive and false-negative detections), they have improved substantially and, coupled with automatic remote monitoring capabilities, they allow for accurate and near-real-time detection of AF burden **(Figure 1)**.^32^

Several studies have taken advantage of these advances in continuous cardiac rhythm monitoring to investigate risk thresholds for AF burden and thromboembolic risk **(Table 1)**.^32–41^ In a secondary analysis of the Mode Selection Trial (MOST), which followed 312 patients with dual-chamber pacemakers for a median of 27 months, atrial high-rate episodes (AHREs) lasting five minutes or more were associated with a significant increase in the risk of death or nonfatal stroke [hazard ratio (HR): 2.79, 95% confidence interval (CI): 1.51–5.15; p = 0.0011].^33^ A similar study using a prospective registry of 725 patients with dual-chamber pacemakers followed for a median of 22 months found that AF episodes lasting between five minutes and 24 hours in length were not associated with a significant increase in thromboembolic risk, but that episodes lasting more than 24 hours were independently associated with a threefold increase in the hazard of thromboembolic events (HR: 3.1, 95% CI: 1.1–10.5; p = 0.044).^34^

The Asymptomatic AF and Stroke Evaluation in Pacemaker Patients and the AF Reduction Atrial Pacing Trial (ASSERT) monitored 2,580 patients aged older than 65 years with dual-chamber pacemakers or ICDs, no history of AF, and a CHADS_2_ score of 2.3 ± 1.1 for a mean of 2.5 years. In this population, subclinical AHREs [defined as an atrial rate of more than 190 beats per minute (bpm) lasting for more than six minutes as detected on the CIED] were discovered in 25% of patients. The annual rate of thromboembolism was 1.7% in patients with AHREs lasting more than six minutes versus 0.7% in patients without such episodes (HR: 1.49, 95% CI: 1.28–4.85; p = 0.007). Additionally, if the longest AHRE was less than 17.7 hours, then the annual rate of stroke or systemic embolism was 1.2%, versus a rate of 4.9% if the longest AHRE recorded was more than 17.7 hours. The total duration but not total number of AHREs correlated with thromboembolic risk, suggesting that the total duration of all AF episodes was more important than AF episode frequency in defining “AF burden” and its relationship with thromboembolic risk.^37^ Notably, the absolute risk of thromboembolism observed in ASSERT was lower than that observed in other studies investigating thromboembolic risk in AF.^42^ It is also difficult to draw direct comparisons between the results from ASSERT and other modern AF trials because none of the ASSERT patients were on anticoagulation at the time of enrollment and only 18% received anticoagulation during follow-up,^37^ while anticoagulation is the standard of care for clinically recognized AF.

**Figure 2:**
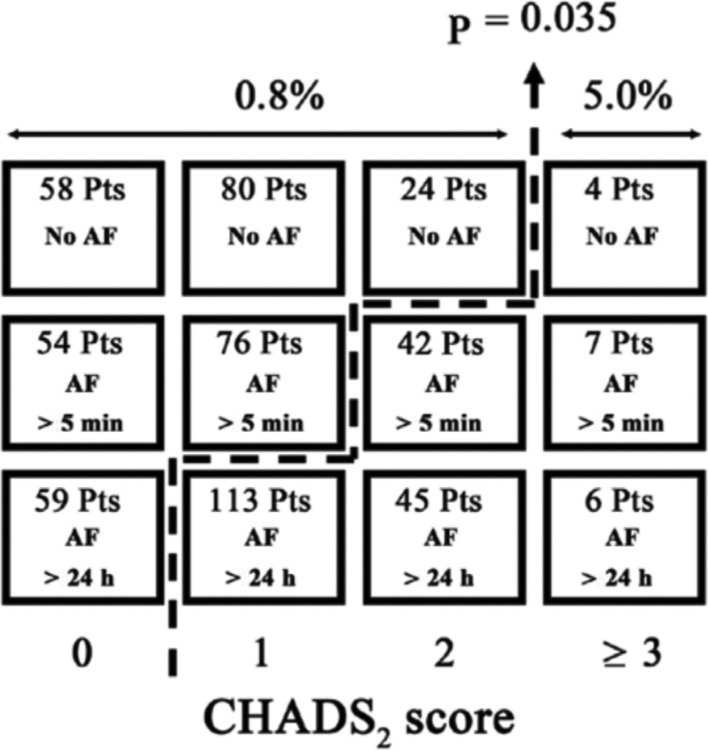
Thromboembolic risk stratified by AF duration and CHADS_2_ score. An assessment of AF burden and thromboembolic risk factors as assessed by the CHADS_2_ score separated patients into two groups with significantly different thromboembolic risks (0.8% versus 5.0%). The columns correspond with CHADS_2_ scores (0, 1, 2, and ≥ 3, respectively) and the rows correspond with AF duration over the course of 24 hours (none, more than five minutes, and 24 hours continuous, respectively). Pts: patients. Reproduced with permission from Botto et al.^35^

Further support for an association between AF burden and thromboembolism was seen in a prospective study of 568 patients after dual-chamber pacemaker implantation. During one year of follow-up, 14 patients (2.5%) experienced a thromboembolic event. A relationship between AF burden and CHADS_2_ score was seen only in patients with intermediate thromboembolic risk (CHADS_2_ score: 1–2), but not in patients with low (CHADS_2_ score: 0) or high (CHADS_2_ score: ≥ 3) scores **(Figure 2)**. Interestingly, thromboembolic risk was similar (5.0%) between patients with a CHADS_2_ score of 1 and continuous AF and those with higher CHADS_2_ scores and any AF burden. Similarly, patients with a CHADS_2_ score of 2 and no AF had a low risk of thromboembolism (0.8%) equivalent to the risk in a patient with a CHADS_2_ score of 1 and short episodes of AF or that in a patient with a CHADS_2_ score of 0 and any AF burden.^35^ These results support the concept that patients who have a low-to-intermediate risk of thromboembolism might be safe without continuous anticoagulation during long periods with a very low burden of AF or during prolonged periods of sinus rhythm.^35^

Newer-generation external cardiac monitors have also demonstrated an association between AF burden and thromboembolism. The Real-world Heart Monitoring Strategy, Evaluation, Treatment Patterns and Health Metrics in AF (RHYTHM) study was a retrospective evaluation of patients with paroxysmal AF who underwent continuous ECG monitoring for up to two weeks using the ZIO® system (iRhythm Technologies, Inc., San Francisco, CA, USA). Each twofold increase in AF burden was associated with a 33% increase in thromboembolism independent of other stroke risk factors.^43^

However, not all studies evaluating AF burden with CIEDs have found a clear association between short AHREs and thromboembolism. The Prospective Study of the Clinical Significance of Atrial Arrhythmias Detected by Implanted Device Diagnostics (TRENDS) study evaluated 2,486 patients with at least one CHADS_2_ risk factor and a pacemaker or ICD with the ability to record AF episodes/AHREs over 1.4 years of follow-up. The study demonstrated increased thromboembolic risk in patients with AHREs lasting more than 5.5 hours versus those with AHREs lasting less than 5.5 hours (2.1% versus 1.1%).^36^ However, among 40 patients who developed a thromboembolic event during the study, only 11 had an AHRE detected within one month prior to thromboembolism. Another study of 560 patients with heart failure and biventricular ICDs noted similar findings. They found that patients with AHREs lasting more than 3.8 hours per day had a significantly increased risk of stroke or systemic embolism (HR: 9.4; p = 0.006) in comparison with those without AHREs. However, only three of the 11 patients with thromboembolism were in AF at the time of stroke.^38^ Even in the ASSERT trial, where there was a clear relationship between subclinical AHREs, a clear temporal association between episodes of AHREs and thromboembolism was not observed: 51 patients had a thromboembolic event but only 26 (51%) of these individuals had subclinical AHREs identified around the time of the event. Furthermore, of these 26 patients with identified AHREs, only four had AHREs within 30 days and only one had AHREs at the time of thromboembolism. Importantly, the CHADS_2_ and CHA_2_DS_2_-VASc scores in the patients who experienced thromboembolism were 2.8 ± 1.1 and 4.5 ± 1.2, respectively.^44^

## Possible explanations for conflicting data in remote monitoring for atrial fibrillation

This conflicting data on the associations between CIED-detected AF burden/AHREs and the timing of thromboembolic events likely stems from several limitations within these studies. First, stroke diagnosis was often defined by clinical examination and not magnetic resonance imaging due to the presence of CIEDs in these patients.^32^ This not only limited the accuracy of stroke diagnosis but also impaired the adjudication of the mechanism of the stroke (thromboembolic versus other). Furthermore, those with very high CHADS_2_ and CHA_2_DS_2_-VASc scores make up an overall sicker population with elevated rates of stroke, even without AF.^45^ This again raises the possibility that, in some patients, atrial stasis from AF may not be the primary driver for AF-related thromboembolism; perhaps, the presence of AF is a marker for other pro-inflammatory and potentially hypercoagulable states that then cause thromboembolism.^6^ In these cases and, in certain patients with particularly elevated thromboembolic risk factors, AF burden may not be the critical determinant for thromboembolic risk.

## Future studies investigating anticoagulation for atrial high-rate episodes

Although multiple studies have demonstrated an association between short AHREs and increased thromboembolic risk, it is not clear how to manage patients who only have subclinical AHREs detected on CIEDs (excluding those with cryptogenic stroke where it is accepted that anticoagulation would be warranted). As noted above, there has not been a clear and consistent temporal association between these subclinical AHREs and thromboembolism and further investigations into whether or not treatment of these episodes with anticoagulation are planned.

The Apixaban for the Reduction of Thromboembolism in Patients with Device-detected Subclinical AF (ARTESiA) trial (NCT01938248) is a prospective, multicenter, double-blind, randomized controlled trial recruiting patients with any subclinical AF (defined as an AF episode lasting more than six minutes but less than 24 hours) on a CIED (eg, pacemaker, ICD, ILR) and additional stroke risk factors as defined by the CHA_2_DS_2_-VASc criteria. Notably, patients with prior documented clinical AF or long episodes of subclinical AF will be excluded and patients with prior thromboembolic events will not be excluded from enrollment, respectively. Anticipated enrollment is approximately 4,000 patients. Subjects will be randomized in a 1:1 ratio to aspirin or apixaban in a blinded manner. The primary endpoint of the study is a composite of stroke or systemic embolism.^46^

Similarly, the Non-vitamin K Antagonist Oral Anticoagulants in Patients with AHREs (NOAH-AFNET 6) trial (NCT02618577) is a randomized study comparing the composite outcome of stroke, systemic embolism, and cardiovascular death in patients with subclinical AF (episodes lasting more than six minutes but without clinically detected AF on a 12-lead ECG) noted on a pacemaker or ICD and additional thromboembolic risk factors. Patients will be randomized to receive treatment with edoxaban or no anticoagulation. Estimated enrollment is approximately 3,400 patients.^47^

## Remote monitoring to guide anticoagulation in patients with atrial fibrillation—“tailored anticoagulation”

Although our understanding of the mechanistic links between AF burden and stroke remain incomplete, based on the above concepts, several trials have aimed to utilize advances in remote monitoring to help guide dynamic use of anticoagulation based on a patient’s AF burden (“tailored anticoagulation”). The concept of tailored anticoagulation is therefore based on the idea that, in select patients with low-to-moderate thromboembolic risk factors, and during prolonged periods of sinus rhythm or in the setting of a very low burden of AF, the risk of thromboembolism is sufficiently low such that continuous anticoagulation is not warranted and, that, during these periods, the patient is exposed to the risks of continuous anticoagulation without a significant benefit in reducing AF-related thromboembolism. Based on the use of DOACs with an onset of action in hours instead of in days as would be expected with warfarin and, with the availability of CIEDs with remote monitoring capabilities, it should therefore be possible to anticoagulate patients promptly around episodes of AF while avoiding the need for anticoagulation when AF is not present.

The Randomized Trial of Anticoagulation Guided by Remote Rhythm Monitoring in Patients With ICD and Resynchronization Devices (IMPACT) multicenter study was the first investigation into tailored anticoagulation. This study did not require a diagnosis of AF for enrollment and also allowed the inclusion of patients with any CHADS_2_ score, including high-risk individuals. As a result, the burden of AF, which prompted the initiation of anticoagulation with warfarin (as the study took place before the widespread availability of DOACs), and the duration of anticoagulation use after AF diagnosis varied based on the CHADS_2_ score. Unfortunately, the IMPACT study was discontinued prematurely due to an absence of benefit.^48^ In retrospect, this study likely failed due to low rates of AF (only 264 out of 2,718 patients had AF during the study); low rates of thromboembolism (only 69 patients experienced a thromboembolic event); and the fact that warfarin was the primary method of anticoagulation, with a suboptimal time in therapeutic range of approximately 59%.^49^

More recent pilot studies using DOACs in low- to-intermediate risk patients with a low burden of AF, however, have shown more promising results. The Rhythm Evaluation for Anticoagulation with Continuous Monitoring (REACT.COM) pilot study was a multicenter single-arm study that enrolled 59 patients (mean age: 67 years ± eight years, 75% male, mean CHADS_2_ score: 1.3 ± 0.5) with implanted Reveal XT™ (Medtronic, Minneapolis, MN, USA) ILRs; nonpermanent AF on a DOAC (apixaban, dabigatran, or rivaroxaban); and a CHADS_2_ score of 1 to 2. Patients could only be enrolled if they had no episodes of AF lasting more than one hour in the two months prior to enrollment. Time on anticoagulation and rates of bleeding and stroke were assessed. Patients made daily remote transmissions using their ILR to assess for AF episodes. After a 60-day run-in with no AF episodes lasting one hour or more, anticoagulation was discontinued and aspirin was started. DOACs were continued or restarted for 30 days following any AF episodes lasting one hour or more.

During 466 ± 131 mean days of follow-up, there were 24,004 ILR transmissions, representing 98.7% compliance with the study protocol. A total of 35 AF episodes lasting one hour or longer occurred in 18 (31%) patients, resulting in 1,472 days on anticoagulation. This represented a 94% reduction in the time on anticoagulation as compared with continuous anticoagulation regardless of AF burden. Two traumatic major bleeds occurred during the trial, both of which happened while patients were on aspirin therapy alone. No strokes or deaths occurred. One “definite” and two “possible” transient ischemic attacks were observed; all three of these patients were not on anticoagulation at the time of their respective neurologic events and all three had no significant AF episodes around the time of diagnosis.^50^

The Tailored Anticoagulation for Noncontinuous AF (TACTIC-AF) pilot study was a similar multicenter randomized controlled study of remote monitoring via dual-chamber pacemakers and ICDs to allow for the intermittent use of DOACs in patients with paroxysmal AF and a CHADS_2_ score < 3. Inclusion criteria included a low burden of AF (< 30 minutes per day and less than six continuous minutes per episode for ≥ 30 days before enrollment as documented on pacemaker or ICD). Patients were randomized to a device-tailored treatment arm or control arm, the latter of which was continuous anticoagulation regardless of AF burden. Those assigned to device-tailored treatment sent twice weekly and AF-alert transmissions to their physicians. Patients were able to discontinue anticoagulation after 30 days of freedom from both a single AF episode of six minutes or more and a total daily AF burden of six hours or more. The tailored anticoagulation arm experienced a 74.6% reduction in time on anticoagulation as compared with standard of care with no reported transient ischemic attacks or strokes. There were two hemorrhagic events each in the control arm (n = 16) and the interventional arm (n = 48), although none of these four patients were on anticoagulation at the time of bleeding.

The results of the REACT.COM and TACTIC-AF pilot studies demonstrate the feasibility and overall safety of tailored anticoagulation in patients with a low burden of AF and low-to-intermediate thromboembolic risk factors. A recent analysis of the REACT.COM data has also demonstrated that tailored anticoagulation can be cost-effective.^51^ It is important to note that these two studies were very small and underpowered to detect adverse outcomes such as stroke and that larger randomized controlled trials are needed before tailored anticoagulation can be adopted into routine clinical practice. Further study of tailored anticoagulation is warranted.

## Limitations of remote monitoring for atrial fibrillation and tailored anticoagulation

Current-generation pacemakers and ICDs primarily detect AF by measuring the rate of atrial activation at the position of the right atrial lead and comparing it with a cutoff (usually approximately 190–200 bpm). Rate-related detection is utilized because localized right atrial activation during AF can often be quite regular, even when the surface ECG demonstrates true AF. As a result, all detected AHREs may not represent AF; some could represent organized atrial tachycardias/flutters or other reentrant supraventricular tachycardias. Current-generation ILRs (and some newer single-chamber ICDs) do not have the ability to assess atrial activity and therefore rely on irregularity of RR intervals to define episodes of AF.^21^ This can result in both false positives (due to frequent atrial/ventricular ectopy) or false negatives (if ventricular rates are relatively regular or if the patient is in a more organized atrial flutter without variable atrioventricular conduction). Noise, electromagnetic interference, or oversensing can also cause false detection of AHREs. Therefore, due to these limitations, in general, AHRE electrograms need to be adjudicated to ensure that they are consistent with AF and not another arrhythmia or a false-positive detection. As future technology and detection algorithms improve, review/adjudication of electrograms will likely become less important. Additionally, the Reveal XT™ (Medtronic, Minneapolis, MN, USA) ILR used in the REACT.COM trial can take up to two minutes to define an episode as AF, depending on how irregular it is. Some short episodes of AF may therefore be missed by ILRs versus by pacemakers or ICDs.

Although the cost-effectiveness of tailored anticoagulation in the REACT.COM study has been shown to be favorable,^51^ tailored anticoagulation is likely associated with significant indirect costs that have not yet been well-quantified. These indirect costs are primarily related to the time and additional personnel required to ensure that all patients are transmitting device data on a regular basis and that their devices are actively communicating with the clinic, with the need to quickly inform patients if they have any AF detected, and with the time required for clinicians to review electrograms to confirm AHRE detections represent AF. However, as AF detection algorithms improve and as CIEDs increase integration with smartphones and other personal device applications, these costs are expected to decrease over time.

## Conclusion/future directions

Despite the high prevalence of AF and a clear benefit associated with anticoagulation in patients with clinically manifest AF, it remains unclear how to optimally treat patients with short, subclinical device-detected AHREs. The optimal cutoff point for treating these episodes also remains unknown. Data suggest, however, that continuous AF episodes lasting more than a few hours but less than 48 hours are associated with increased thromboembolic risk, with the absolute risk likely modulated by individual patient risk factors. Studies investigating the treatment of these short, subclinical AHREs are currently enrolling patients.

The currently accepted approach to anticoagulation in AF is incomplete and contradictory. AF burden does not factor in the decision to anticoagulate for AF, yet AF episodes lasting up to 48 hours are thought to be associated with a thromboembolic risk that is low enough to defer transesophageal echocardiogram prior to cardioversion. Recent data, however, have challenged this cornerstone principle of AF management. The availability of continuous atrial arrhythmia monitoring with automatic remote transmission of arrhythmia data and rapidly acting oral anticoagulants has allowed the concept of tailored anticoagulation, wherein some lower-risk patients with a relatively low burden of AF may be able to safely start and stop oral anticoagulation based on their AF burden, to emerge. Preliminary data suggest that tailored anticoagulation is feasible and safe. Further studies will be required, however, before it can be adopted into routine clinical practice.

## Figures and Tables

**Table 1:** Studies Demonstrating a Correlation Between AF Burden/AHREs and Stroke or Systemic Embolism

Study	Number of Patients	Study Type	AF Monitoring	Follow-up	Outcomes	
Glotzer et al.^33^	312	Secondary analysis of multicenter RCT (MOST)	Dual-chamber PPM	27 months (median)	•	Ten patients (3.2%) developed stroke
					•	AHREs lasting five minutes or longer were associated with an HR of 2.79 for death or nonfatal stroke (p = 0.0011)
Capucci et al.^34^	725	Prospective registry (AT500 Registry)	Dual-chamber PPM	22 months (median)	•	Fourteen patients (1.9%) developed a thromboembolic event (11 were stroke or TIA)
					•	AF episodes lasting > 24 hours were associated with adjusted HR of 3.1 for thromboembolic event (p = 0.044)
					•	AF episodes lasting between five minutes and 24 hours were not associated with a significant increase in thromboembolic risk
Botto et al.^35^	568	Prospective observational study	Dual-chamber PPM	1 year	•	Fourteen patients (2.5%) developed stroke or systemic embolism
					•	When patients were stratified into multiple groups based on CHADS_2_ score (0, 1, 2, or ≥ 3), and duration of AF episodes (< 5 minutes, 5 minutes to 24 hours, and 24 hours continuously) was more than 24 hours, this allowed the selection of two patient populations with significantly different annual rates of thromboembolic events (0.8% versus 5.0%)
					•	Patients with a high CHADS_2_ score and any burden of AF and patients with a low CHADS_2_ score and a high burden of AF, respectively, had increased rates of thromboembolism
Glotzer et al.^36^	2,486	Prospective observational study (TRENDS)	Dual-chamber PPM or ICD	1.4 years (mean)	•	Annual thromboembolic risk was 1.1% in patients with no AF, 1.1% in patients with AF episodes lasting < 5.5 hours (low burden), and 2.1% in patients with AF episodes lasting ≥ 5.5 hours (high burden)
					•	No statistically significant differences in thromboembolic events between the no AF, low-burden, and high-burden groups were found, although the p value was of borderline significance in a comparison between high burden and zero burden (HR: 2.20; p = 0.06)
					•	Thirty-day cumulative AF burden ≥ 10.8 hours showed a trend towards an association with an increased risk of thromboembolism (HR: 2.22; p = 0.06)
Healey et al.^37^	2,580	Primary analysis of RCT (ASSERT)	Dual-chamber PPM or ICD	2.5 years (mean)	•	The annual rate of thromboembolism was 1.69% in patients with atrial tachyarrhythmia episodes lasting more than six minutes as compared with 0.69% in patients with episodes lasting less than six minutes (HR: 1.76; p = 0.05)
					•	For the longest atrial tachyarrhythmia < 17.7 hours, the annual rate of stroke or systemic embolism was 1.2%
					•	For the longest atrial tachyarrhythmia > 17.7 hours, the annual rate of stroke or systemic embolism was 4.9%
Shanmugan et al.^38^	560	Secondary analysis of two prospective, multicenter, observational studies (Home CARE and everesT)	Biventricular PPM or ICD	370 days (median)	•	Eleven patients (2%) experienced thromboembolic events
					•	AHREs ≥ 3.8 hours/day were associated with an HR of 9.4 (p = 0.006) for stroke or systemic embolism as compared with in patients without arrhythmia
Boriani et al.^39^	10,016	Pooled analysis of three prospective studies (TRENDS, PANORAMA, and Italian ClinicalService^®^ Registry)	PPM or ICD with atrial lead	24 months (median)	•	Ninety-five patients (0.39%/year) experienced stroke or systemic embolism
					•	AF burden was independently associated with thromboembolism at multiple cutoff points
					•	AF episodes lasting more than one hour were associated with an HR of 2.11 (p = 0.008) for ischemic stroke
					•	AF episodes lasting more than five minutes were associated with an HR of 1.76 (p = 0.041) for ischemic stroke
					•	For every hour of AF in a 24-hour period, the relative risk for stroke increased by 3%
Witt et al.^40^	394	Prospective, single-center, observational study of CRT patients in Denmark	Biventricular PPM or ICD	4.6 years (median)	•	Thirty patients (7.6%) experienced thromboembolic events
					•	Patients with AHREs lasting more than six minutes were associated with a 3.1%/year risk of thromboembolism as compared with a 1.4%/year risk in patients with no AHREs or AHREs lasting less than six minutes in duration
					•	The presence of AHREs lasting more than six minutes was associated with an HR of 2.30 (p = 0.028) for thromboembolism
					•	Thromboembolic risk of AHREs persisted after adjustment for CHA_2_DS_2_-VASc score (HR: 2.52; p = 0.015)
					•	AHREs lasting longer than 24 hours were associated with an even higher thromboembolic risk (HR: 3.13; p = 0.023)
Swiryn et al.^41^	5,379	Prospective, multi-center registry (RATE)	Dual chamber PPM or ICD	2 years	•	Defined AHRE as three or more premature atrial contractions; “short” episodes had both onset and offset within the same electrogram strip, while “long” episodes had onset and offset that were not on the same electrogram strip. No specific time definitions for AHREs were used, with “long” episodes usually lasting > 20 seconds.
					•	“Short” AHREs were not associated with an increased risk of stroke/TIA (HR: 0.87; p = 0.51)
					•	“Long” AHREs were associated with an increased risk of stroke/TIA (HR: 1.51; p = 0.03)

AF: atrial fibrillation; AHRE: atrial high-rate episode; RCT: randomized controlled trial; PPM: permanent pacemaker; HR: hazard ratio; TIA: transient ischemic attack; ICD: implantable cardioverter-defibrillator; CRT: cardiac resynchronization therapy. Adapted with permission from Zimetbaum et al.^32^

## References

[1] Benjamin EJ, Blaha MJ, Chiuve SE (2017). Heart disease and stroke statistics—2017 update: a report from the American Heart Association. Circulation..

[2] Chugh SS, Havmoeller R, Narayanan K (2014). Worldwide epidemiology of atrial fibrillation: a Global Burden of Disease 2010 Study. Circulation..

[3] Colilla S, Crow A, Petkun W, Singer DE, Simon T, Liu X (2013). Estimates of current and future incidence and prevalence of atrial fibrillation in the U.S. adult population. Am J Cardiol..

[4] Fuster V, Ryden LE, Cannom DS (2006). ACC/AHA/ESC 2006 Guidelines for the Management of Patients with Atrial Fibrillation: a report of the American College of Cardiology/American Heart Association Task Force on Practice Guidelines and the European Society of Cardiology Committee for Practice Guidelines (Writing Committee to Revise the 2001 Guidelines for the Management of Patients With Atrial Fibrillation): developed in collaboration with the European Heart Rhythm Association and the Heart Rhythm Society. Circulation..

[5] Hart RG, Pearce LA, Aguilar MI (2007). Meta-analysis: antithrombotic therapy to prevent stroke in patients who have nonvalvular atrial fibrillation. Ann Intern Med..

[6] Kamel H, Okin PM, Elkind MSV, Iadecola C (2016). Atrial fibrillation and mechanisms of stroke: time for a new model. Stroke..

[7] January CT, Wann LS, Alpert JS (2014). 2014 AHA/ACC/HRS guideline for the management of patients with atrial fibrillation: a report of the American College of Cardiology/American Heart Association Task Force on practice guidelines and the Heart Rhythm Society. Circulation..

[8] Wood W (1814). Letter enclosing the history and dissection of a case in which a foreign body was found within the heart. Edinb Med Surg J..

[9] Harvey EA, Levine SA (1930). A study of uninfected mural thrombi of the heart. Am J M Sci..

[10] Daley R, Mattingly TW, Holt CL, Bland EF, White PD (1951). Systemic arterial embolism in rheumatic heart disease. Am Heart J..

[11] Wolf PA, Dawber TR, Thomas HE, Kannel WB (1978). Epidemiologic assessment of chronic atrial fibrillation and risk of stroke: the Framingham study. Neurology..

[12] Petersen P, Godtfredsen J (1986). Embolic complications in paroxysmal atrial fibrillation. Stroke..

[13] Stroke Prevention in Atrial Fibrillation Study (1991). Final results. Circulation..

[14] Petersen P, Boysen G, Godtfredsen J, Andersen ED, Andersen B (1989). Placebo-controlled, randomised trial of warfarin and aspirin for prevention of thromboembolic complications in chronic atrial fibrillation. The Copenhagen AFASAK study. Lancet..

[15] Singer DE, Hughes RA, Boston Area Anticoagulation Trial for Atrial Fibrillation Investigators (1990). The effect of low-dose warfarin on the risk of stroke in patients with nonrheumatic atrial fibrillation. N Engl J Med..

[16] Connolly SJ, Laupacis A, Gent M, Roberts RS, Cairns JA, Joyner C (1991). Canadian atrial fibrillation anticoagulation (CAFA) study. J Am Coll Cardiol..

[17] EAFT (European Atrial Fibrillation Trial) Study Group (1993). Secondary prevention in non-rheumatic atrial fibrillation after transient ischaemic attack or minor stroke. Lancet..

[18] Gage BF, Waterman AD, Shannon W, Boechler M, Rich MW, Radford MJ (2001). Validation of clinical classification schemes for predicting stroke: results from the National Registry of Atrial Fibrillation. JAMA..

[19] Cardio-Thoracic S, Camm AJ, European Heart Rhythm A, European Association for Cardio-Thoracic S (2010). Guidelines for the management of atrial fibrillation: the Task Force for the Management of Atrial Fibrillation of the European Society of Cardiology (ESC). Eur Heart J..

[20] Piccini JP, Singer DE (2012). Putting risk prediction in atrial fibrillation into perspective. Eur Heart J..

[21] Hess PL, Healey JS, Granger CB (2017). The role of cardiovascular implantable electronic devices in the detection and treatment of subclinical atrial fibrillation: a review. JAMA Cardiol..

[22] Argulian E, Conen D, Messerli FH (2015). Misconceptions and facts about atrial fibrillation. Am J Med..

[23] Weigner MJ, Caulfield TA, Danias PG, Silverman DI, Manning WJ (1997). Risk for clinical thromboembolism associated with conversion to sinus rhythm in patients with atrial fibrillation lasting less than 48 hours. Ann Intern Med..

[24] Patten M, Maas R, Karim A, Muller HW, Simonovsky R, Meinertz T (2006). Event-recorder monitoring in the diagnosis of atrial fibrillation in symptomatic patients: subanalysis of the SOPAT trial. J Cardiovasc Electrophysiol..

[25] Verma A, Champagne J, Sapp J (2013). Discerning the incidence of symptomatic and asymptomatic episodes of atrial fibrillation before and after catheter ablation (DISCERN AF): a prospective, multicenter study. JAMA Intern Med..

[26] Hindricks G, Piorkowski C, Tanner H (2005). Perception of atrial fibrillation before and after radiofrequency catheter ablation: relevance of asymptomatic arrhythmia recurrence. Circulation..

[27] Hohnloser SH, Pajitnev D, Pogue J (2007). Incidence of stroke in paroxysmal versus sustained atrial fibrillation in patients taking oral anticoagulation or combined antiplatelet therapy: an ACTIVE W substudy. J Am Coll Cardiol..

[28] Hart RG, Pearce LA, Rothbart RM, McAnulty JH, Asinger RW, Halperin JL (2000). Stroke with intermittent atrial fibrillation: incidence and predictors during aspirin therapy. J Am Coll Cardiol..

[29] AFFIRM First Antiarrhythmic Drug Substudy Investigators (2003). Maintenance of sinus rhythm in patients with atrial fibrillation: an AFFIRM substudy of the first antiarrhythmic drug. J Am Coll Cardiol..

[30] Hohnloser SH, Pajitnev D, Pogue J (2007). Incidence of stroke in paroxysmal versus sustained atrial fibrillation in patients taking oral anticoagulation or combined antiplatelet therapy: an ACTIVE W Substudy. J Am Coll Cardiol..

[31] Al-Khatib SM, Thomas L, Wallentin L (2013). Outcomes of apixaban vs. warfarin by type and duration of atrial fibrillation: results from the ARISTOTLE trial. Eur Heart J..

[32] Zimetbaum P, Waks JW, Ellis ER, Glotzer TV, Passman RS (2014). Role of atrial fibrillation burden in assessing thromboembolic risk.. Circ Arrhythm Electrophysiol..

[33] Glotzer TV, Hellkamp AS, Zimmerman J (2003). Atrial high rate episodes detected by pacemaker diagnostics predict death and stroke: report of the Atrial Diagnostics Ancillary Study of the MOde Selection Trial (MOST). Circulation..

[34] Capucci A, Santini M, Padeletti L (2005). Monitored atrial fibrillation duration predicts arterial embolic events in patients suffering from bradycardia and atrial fibrillation implanted with antitachycardia pacemakers.. J Am Coll Cardiol..

[35] Botto GL, Padeletti L, Santini M (2009). Presence and duration of atrial fibrillation detected by continuous monitoring: crucial implications for the risk of thromboembolic events. J Cardiovasc Electrophysiol..

[36] Glotzer TV, Daoud EG, Wyse DG (2009). The relationship between daily atrial tachyarrhythmia burden from implantable device diagnostics and stroke risk: the TRENDS study. Circ Arrhythm Electrophysiol..

[37] Healey JS, Connolly SJ, Gold MR (2012). Subclinical atrial fibrillation and the risk of stroke. N Engl J Med..

[38] Shanmugam N, Boerdlein A, Proff J (2012). Detection of atrial high-rate events by continuous home monitoring: clinical significance in the heart failure-cardiac resynchronization therapy population. Europace..

[39] Boriani G, Glotzer TV, Santini M (2014). Device-detected atrial fibrillation and risk for stroke: an analysis of >10,000 patients from the SOS AF project (Stroke preventiOn Strategies based on Atrial Fibrillation information from implanted devices). Eur Heart J..

[40] Witt CT, Kronborg MB, Nohr EA, Mortensen PT, Gerdes C, Nielsen JC (2015). Early detection of atrial high rate episodes predicts atrial fibrillation and thromboembolic events in patients with cardiac resynchronization therapy. Heart Rhythm..

[41] Swiryn S, Orlov MV, Benditt DG (2016). Clinical implications of brief device-detected atrial tachyarrhythmias in a cardiac rhythm management device population: results from the Registry of Atrial Tachycardia and Atrial Fibrillation Episodes. Circulation..

[42] Connolly S, Pogue J, Hart R (2006). Clopidogrel plus aspirin versus oral anticoagulation for atrial fibrillation in the Atrial fibrillation Clopidogrel Trial with Irbesartan for prevention of Vascular Events (ACTIVE W): a randomised controlled trial. Lancet..

[43] Go A Burden of atrial fibrillation and thromboembolism risk: the RHYTHM (Real-world Heart Monitoring Strategy, Evaluation, Treatment Patterns and Health Metrics in Atrial Fibrillation) study. Paper presented at: Heart Rhythm Society Scientific Sessions; May 6, 2016. San Francisco, CA.

[44] Brambatti M, Connolly SJ, Gold MR (2014). Temporal relationship between subclinical atrial fibrillation and embolic events. Circulation..

[45] Welles CC, Whooley MA, Na B, Ganz P, Schiller NB, Turakhia MP (2011). The CHADS2 score predicts ischemic stroke in the absence of atrial fibrillation among subjects with coronary heart disease: data from the Heart and Soul Study. Am Heart J..

[46] Lopes RD, Alings M, Connolly SJ (2017). Rationale and design of the Apixaban for the Reduction of Thrombo-Embolism in Patients With Device-Detected Sub-Clinical Atrial Fibrillation (ARTESiA) trial. Am Heart J..

[47] Kirchhof P, Blank BF, Calvert M (2017). Probing oral anticoagulation in patients with atrial high rate episodes: Rationale and design of the Non-vitamin K antagonist Oral anticoagulants in patients with Atrial High Rate episodes (NOAH-AFNET 6) trial. Am Heart J..

[48] Ip J, Waldo AL, Lip GY (2009). Multicenter randomized study of anticoagulation guided by remote rhythm monitoring in patients with implantable cardioverter-defibrillator and CRT-D devices: rationale, design, and clinical characteristics of the initially enrolled cohort: the IMPACT study. Am Heart J..

[49] Martin DT, Bersohn MM, Waldo AL (2015). Randomized trial of atrial arrhythmia monitoring to guide anticoagulation in patients with implanted defibrillator and cardiac resynchronization devices. Eur Heart J..

[50] Passman R, Leong-Sit P, Andrei AC (2016). Targeted anticoagulation for atrial fibrillation guided by continuous rhythm assessment with an insertable cardiac monitor: the Rhythm Evaluation for Anticoagulation With Continuous Monitoring (REACT.COM) pilot study. J Cardiovasc Electrophysiol..

[51] Steinhaus DA, Zimetbaum PJ, Passman RS, Leong-Sit P, Reynolds MR (2016). Cost-effectiveness of implantable cardiac monitor-guided intermittent anticoagulation for atrial fibrillation: an analysis of the REACT.COM pilot study. J Cardiovasc Electrophysiol..

[52] Zimetbaum P, Ellis ER, Waks JW, Passman RS (2013). The importance of atrial fibrillation burden and the origin of device-tailored anticoagulation. Pacing Clin Electrophysiol..

